# The Effects of Artificial Sweeteners on Intestinal Nutrient-Sensing Receptors: Dr. Jekyll or Mr. Hyde?

**DOI:** 10.3390/life14010010

**Published:** 2023-12-20

**Authors:** Edit Posta, Istvan Fekete, Eva Gyarmati, László Stündl, Eva Zold, Zsolt Barta

**Affiliations:** 1 GI Unit, Department of Infectology, Faculty of Medicine, University of Debrecen, Bartok Bela Street 2-26, 4031 Debrecen, Hungary; gyarmati.eva@med.unideb.hu (E.G.); barta@med.unideb.hu (Z.B.); 2 Institute of Food Technology, Faculty of Agricultural and Food Sciences and Environmental Management, University of Debrecen, Böszörményi út 138, 4032 Debrecen, Hungary; feketei@agr.unideb.hu (I.F.); stundl@agr.unideb.hu (L.S.); 3 Doctoral School of Clinical Immunology and Allergology, Faculty of Medicine, University of Debrecen, Nagyerdei Blvd. 98, 4032 Debrecen, Hungary; 4 Department of Clinical Immunology, Institute of Internal Medicine, Faculty of Medicine, University of Debrecen, Móricz Zsigmond Str. 22, 4032 Debrecen, Hungary; zold.eva@med.unideb.hu

**Keywords:** artificial sweeteners, nutrient-sensing receptor, gastrointestinal, IBD, incretin, microbiome

## Abstract

The consumption of artificial and low-calorie sweeteners (ASs, LCSs) is an important component of the Western diet. ASs play a role in the pathogenesis of metabolic syndrome, dysbiosis, inflammatory bowel diseases (IBDs), and various inflammatory conditions. Intestinal nutrient-sensing receptors act as a crosstalk between dietary components, the gut microbiota, and the regulation of immune, endocrinological, and neurological responses. This narrative review aimed to summarize the possible effects of ASs and LCSs on intestinal nutrient-sensing receptors and their related functions. Based on the findings of various studies, long-term AS consumption has effects on the gut microbiota and intestinal nutrient-sensing receptors in modulating incretin hormones, antimicrobial peptides, and cytokine secretion. These effects contribute to the regulation of glucose metabolism, ion transport, gut permeability, and inflammation and modulate the gut–brain, and gut–kidney axes. Based on the conflicting findings of several in vitro, in vivo, and randomized and controlled studies, artificial sweeteners may have a role in the pathogenesis of IBDs, functional bowel diseases, metabolic syndrome, and cancers via the modulation of nutrient-sensing receptors. Further studies are needed to explore the exact mechanisms underlying their effects to decide the risk/benefit ratio of sugar intake reduction via AS and LCS consumption.

## 1. Introduction

The Western diet is characterized by high fat and sugar consumption. This lifestyle factor presents a high risk for intestinal dysbiosis, which leads to leaky gut, metabolic syndrome, and various inflammatory conditions [1]. A high-fat high-sugar diet increases inflammatory markers in inflammatory bowel diseases (IBDs), which are characterized by chronic inflammation of the gastrointestinal tract with relapsing and remitting clinical course. IBDs refer to Crohn’s disease and ulcerative colitis [2]. Because of this, reduced sugar intake has become a popular trend, and the usage of artificial sweeteners has increased in the past few decades [3,4].

In recent years, there have been several studies showing that artificial sweeteners (ASs) may have a role in the increasing prevalence of obesity, metabolic syndrome, cancer, and type 2 diabetes mellitus through the induction of dysbiosis, but the findings of these studies are controversial [5]. It should not be forgotten that there are industrial interests and sponsored studies, which does not make it easy to see clearly in this field. In fact, intestinal microbiota dysbiosis has a key role not only in the pathogenesis of metabolic syndrome [1] but also in inflammatory bowel diseases and other chronic inflammatory conditions. The increasing consumption of artificial sweeteners, e.g., saccharin, has also been found to be positively correlated with the increasing prevalence of IBD [6].

Artificial sweeteners bind to sweet (T1R) taste receptors, and some intense sweeteners can also bind to bitter (T2R) taste receptors [7,8]. Therefore, they may modify the function and effect of these receptors not only in the oral cavity but also in the gastrointestinal system.

In one of the first investigations about taste-sensing receptors in 1999, the gene expression pattern of mammalian taste receptors was described on the fungiform and circumvallate papillae of the tongue [9]. These receptors are expressed on multiple cells in the oral cavity and the gastrointestinal tract. Moreover, the whole body takes part in various metabolic and immunological processes.

**Figure 1:**
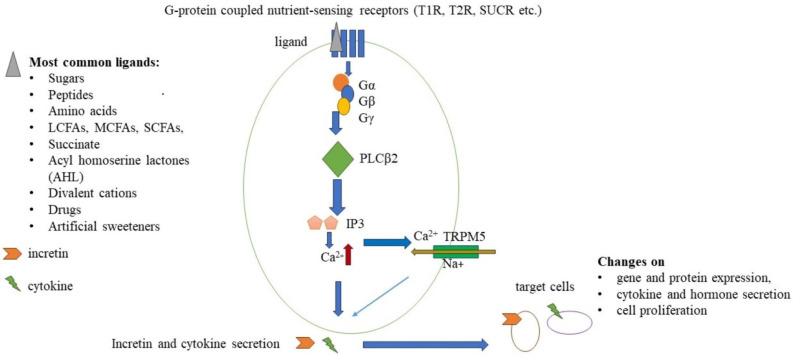
Schematic signaling pathway of nutrient sensing, G−protein coupled receptors. Ligands (grey triangle) bind to nutrient−sensing receptors and coupled G−proteins to activate PLCβ2. IP3 is activated by PLCβ2 and binds to IP3 receptors to cause intracellular Ca^2+^ release (red arrow), which drives the opening of the TRPM5 cation channel and Na^+^ influx (dark yellow arrow). These changes lead to increased incretin and cytokine secretion by enteroendocrine cells. These molecules could have an influence on the gene and protein expressions, various cytokine and hormone secretion, and modified cell proliferation of target cells.

T1R and T2R receptors are members of the G protein-coupled receptor family, and the T1R family consists of three different receptors, while the T2R family has more than 25 members [10]. Umami and sweet agonists can activate specific T1R heterodimers. T1R1/T1R3 responds to L-amino acids such as monosodium glutamate (MSG) [11]. T1R2/T1R3 heterodimers are sensitive to sweet agonists. Moreover, the Tas1R2 gene encodes a single-nucleotide polymorphism (SNP) that affects sweet perception and sugar intake in a BMI-dependent manner (BMI above 25) [12], while polymorphisms of the bitter taste receptor T2R38 result in supertasters for bitterness. It could lead to the avoidance of some vegetables and fruits (e.g., *Brassica* ssp., spinach, bitter citrus, and green tea) and increased consumption of sweet and fatty food, thereby increasing the risk for chronic metabolic and inflammatory diseases [13]. The transient receptor potential cation channel subfamily M member 5 (TRPM5) is a voltage-sensitive, monovalent cation-selective channel. It is activated by elevated intracellular Ca^2+^, which is increased by the signaling of T1R/T2R and other G protein-coupled taste receptors via phospholipase C beta 2 (PLCß2) activation, which plays a key role in this process (Figure 1) [14].

This review aimed to summarize the significance of nutrient-sensing receptors and the possible effects of artificial and low-calorie sweetener consumption on these receptors and their pathways.

## 2. Materials and Methods

Search method: The literature search was conducted using the PubMed and Google Scholar databases with no restrictions on the publication date and country. The following search terms were used alone and in combination: artificial sweeteners, low-calorie sweeteners, sugar alcohols, steviol glycosides, sweet and bitter taste receptors, nutrient-sensing receptors, calcium-sensing receptors, amino acid-sensing receptors, FFAR 1–4, lipid-sensing receptors, gastrointestinal system, inflammatory bowel disease, inflammation, metabolism, metabolic syndrome, type 2 diabetes mellitus, cancer risk, cardiovascular diseases, innate immunity, immunology, microbiome, and allergy. Based on these search terms, relevant articles were identified (backward search) and, in some cases, newer articles cited the original papers, which were then located (forward search).

Main inclusion criteria: Articles had to be written in English, published in a peer-reviewed journal, and without industrial funding. These articles must focus on research on nutrient-sensing receptors, as well as artificial and low-calorie sweeteners and their effects, based on the search terms. Main exclusion criteria: articles that were not written in English, were not published in a peer-reviewed journal, were protocols, or did not match the search terms were excluded.

**Figure 2:**
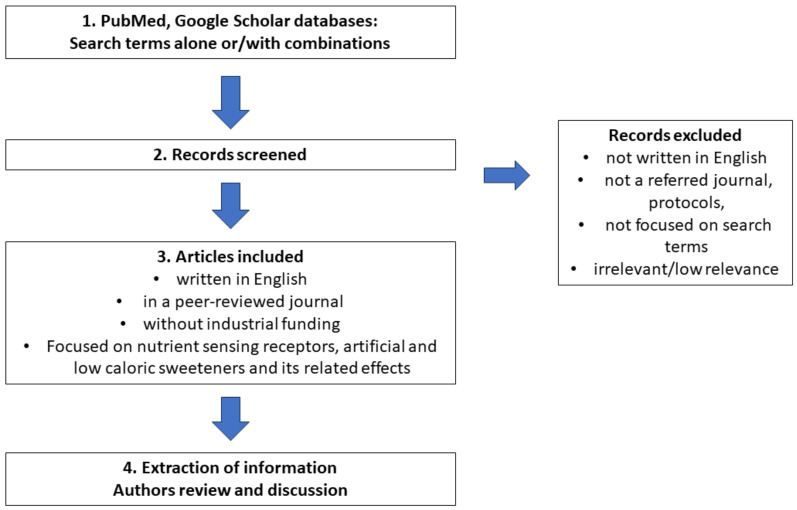
Schematic figure about our review stages.

Extraction of information: Information regarding the molecular pathways, related functions and significance of nutrient-sensing receptors, effects of artificial sweeteners, and study design was extracted from the included articles. The authors independently reviewed each article and extracted the information. In the case of any conflict in interpretation between the authors, a discussion was held and the issue was resolved through consensus (Figure 2).

## 3. The Role of Nutrient-Sensing Receptors outside and inside the Gastrointestinal System

### 3.1. Sweet and Bitter Taste Receptors (T1Rs and T2Rs)

Sweet and bitter taste receptors are expressed throughout the body.

Sweet taste receptors are widely expressed in the brain and the hypothalamic arcuate nucleus. Some neurons are sensitive to higher concentrations of glucose and are referred to as high-glucose-excited (HGE) neurons. These HGE neurons not only express sweet taste receptors but also sodium-glucose-linked transporters (SGLT1 and SGLT3). The exact role of sweet taste receptors in the brain is not known. They may have a role in the regulation of glucose homeostasis, the blood-brain barrier permeability, the circadian rhythm, and feeding behavior based on mouse model studies [15,16].

Interestingly, a previous study found that T2R38 was expressed on the fetal side of the human placenta; the amnion epithelium and the syncytiotrophoblast had strong positivity for T2R38, while on the maternal side, a weak expression signal was detected. T2R38 was also expressed in JEG-3 cells, a human placental cell line [17]. In another study, T2R14 expression was detected immunohistochemically in human syncytiotrophoblast and extravillous trophoblast, while in a human placental cell line, T2R14 agonists (flufenamic acid, chlorhexidine, and diphenhydramine) caused intracellular calcium release. The exact roles of these natural ligands are currently unknown [18].

T2R plays a role in the recognition of bacterial products and host-pathogen interactions. In a systematic review and meta-analysis, the single-nucleotide polymorphisms (SNPs) of four different genes (TAS1R2, TAS2R38, TAS1R3, and GLUT2) were associated with dental caries experience and oral health [19]. In a small but well-designed study, T2R38 polymorphisms were associated with the composition of the oral microbiome of rheumatoid arthritis (RA) patients, but there was no relation with anti-citrullinated protein levels (the most important antibody in RA) [20].

Acyl-homoserine lactone (AHL) molecules are secreted by Gram-negative bacteria such as Pseudomonas aeruginosa. AHLs can activate T2R38 receptors, which are expressed in human sinonasal cilia. This could act as a signal to activate nitrogen oxide synthase-dependent nitrogen oxide production. There are solitary chemosensory cells in the upper airway system, which express T1R and T2R receptors, and the activation of these receptors may lead to antimicrobial peptide secretion (ß-defensin 1 and 2) [21]. This secretion is very rapid, with the majority occurring within 5 min, while TLR-mediated ß-defensin secretion takes hours after the activation of TLRs. This means that the difference between Toll-like receptor (TLR)-mediated secretion of antimicrobial peptides and that mediated by T1R and T2R receptors is the timescale. Activation of T1Rs inhibits T2R-dependent calcium signaling in human solitary chemosensory cells. Interestingly, in previous research, an elevated glucose concentration was measured in the airway surface liquid of patients with rhinosinusitis but without diabetes mellitus in contrast to healthy controls. This could lead to sufficient antimicrobial peptide secretion. In contrast, during acute infections, bacteria consume glucose locally, so T1Rs cannot be activated [22,23]. In the urinary tract, there are chemosensory brush cells, which express T1Rs and T2Rs. These cells can activate the bladder detrusor muscle and respond to uropathogenic *E. coli* strains, suggesting that they have a role in antimicrobial innate immunity [23].

T2R and T1R are expressed in human neutrophil granulocytes, T cells, and B cells. T2R38 is expressed on lymphocytes and may have a role in adaptive immune responses and migration. It is also expressed on skin-infiltrating lymphocytes in atopic dermatitis [24]. The sweet taste receptor T1R3 is expressed on T and B lymphocytes. Saccharin can induce neutrophil migration, whereas lactisole, which is a selective inhibitor of T1R3, can inhibit neutrophil migration [25]. T2R38 is expressed on myeloid cells but on macrophages rather than monocytes. Previous in vitro studies have suggested that it is activated by a Gram-negative bacterial product, AHL-12, and based on a human in vitro investigation, T2R38 may play a role in the detection of bacterial biofilms [26]. T2R receptor activation enhances the phagocytosis of human macrophages in vitro via the elevation of intracellular calcium release, and endothelial nitric oxide synthase (eNOS) activation via the cyclic GMP (cGMP) pathways [27]. In an animal model of allergic asthma, treatment with T2R agonists (chloroquine and quinine) inhibits mucus secretion, allergen-induced airway inflammation, remodeling, MMP release, and neutrophil chemotaxis in a dose-dependent manner in mice [28]. Moreover, T2R agonists inhibit immunoglobulin E (IgE)-dependent mast cell activation and decrease histamine and/or prostaglandin D2 release in mice [29]. In an in vitro model of diabetic nephropathy, T1R3 receptors took part in reactive oxygen species-NLRP3 inflammasome activation after high-glucose treatment [30].

Based on these studies, sweet and bitter receptors not only take part in innate immunity, leukocyte migration, bacterial recognition, and chemotaxis but may also have a role in allergic and infectious diseases.

These receptors are expressed not only on immune cells of the gastrointestinal mucosa but also on multiple cell types, including enteroendocrine cells, tuft cells, goblet cells, and Paneth cells [14]. These cells can detect antimicrobial peptides through TLRs, and this leads to the secretion of cytokines and peptide hormones.

Enteroendocrine cells secrete incretins, which are variable peptide hormones (i.e., ghrelin, gastrin, leptin, somatostatin, cholecystokinin, glucose-dependent insulinotropic polypeptide (GIP), and glucagon-like peptide-1 (GLP-1)) along the gastrointestinal tract, depending on their localization (Table 1) [31]. Not only T1Rs detect luminal glucose concentration and regulate incretin hormone secretion, but T2Rs may have a role in the pathogenesis of diabetes mellitus. Four single-nucleotide polymorphisms (SNPs) of T2Rs show an association with type 2 diabetes mellitus [32]. In a previous study, the expression of T1R2 taste receptors was inversely regulated by glucose levels in healthy controls (*n* = 14), but not in type 2 diabetes mellitus patients (*n* = 13), which means that sweet taste receptor regulation is altered [33]. These hormones could modulate innate and adaptive immune responses, as well as intestinal permeability, and act as cytokines. In clinical studies, the serum levels of the above hormones show significant changes in inflammatory bowel diseases [34,35,36,37]. The SGLT1 glucose transporter is essential in glucose metabolism regulation and is expressed by absorptive enterocytes. Its expression is regulated by EEC-secreted peptide hormones such as GLP-1,2. [38]. GLP-1,2 and GIP stimulates pancreatic ß-islet insulin secretion, inhibit gastric emptying, and reduce food ingestion. GLPs are rapidly degraded by dipeptidyl peptidase-4 (DPP-4), and thus, incretin hormones have a short half-life. GLP-agonist and DPP4 inhibitor antidiabetic drugs affect the incretin inflammatory axis, and the role of intestinal inflammation and immune regulation is debated. A question arises as to what the impact on the risk and course of inflammatory bowel diseases and the risk of cancer might be, so further investigations and clinical studies are needed [39].

Chemosensory tuft cells play a key role in parasite and helminth infections in initiating type 2 immunity via IL-25 secretion and innate lymphoid cell type 2 (ILC2) stimulation. T1R3 receptor regulates the homeostasis of tuft cell abundance. In Tas1R3−/− knockout mice, not only was a decreased tuft cell number observed, but this number was only partially compensated by succinate treatment [40,41].

Tuft cells participate in some viral infections as a direct or indirect target of infective agents (e.g., in the case of West Nile virus, they have an immunomodulatory effect in the pathogenesis of this infection) [42]. Succinate, which is a metabolite of some symbiotic bacteria and helminths, acts as a ligand on succinate receptor 1 (SUCR1) and in the TRPM5-dependent pathway [43]. Tuft cells have a possible role in the pathogenesis of inflammatory bowel diseases based on a few studies. Decreased tuft cell number was observed in the colonic biopsies of patients suffering from quiescent ulcerative colitis [44]. Moreover, a reduced tuft cell number was detected in the ileal biopsies of patients with Crohn’s disease, and their number was negatively correlated with the level of inflammation [43].

Goblet cells are expressed along the small and large intestines and produce mucin to protect the intestinal surface of the epithelium against bacterial invasion. Their number is increased in the distal part of the gut and correlates with the number of bacteria and the expression of T2R131 in mice [45].

Paneth cells are located in Lieberkühn crypts and secrete antimicrobial peptides and alpha-defensins to control the local microbiome. In ileal Crohn’s disease, Paneth cell number and the secretion of defensins are decreased. T2R43 and T2R10 are detected in the human goblet and Paneth cells, and bitter agonists could cause intracellular Ca^2+^ release in these cells, which means that T2Rs may have a functional role. Moreover, in previous research, treatment with the bitter agonist, denatonium benzoate, induced the expression of antimicrobial peptide and alpha defensin-5 protein in the Paneth cells of patients with obesity. This effect was not seen in healthy controls, while T2R mRNA and protein expression levels were the same in in vitro experiments. The Paneth cells of patients with obesity were more sensitive to bitter-induced degranulation, which altered microbial growth [46,47]. There was also relative goblet cell depletion and defective mucous secretion in these cells in active ulcerative colitis [48].

According to several in vitro studies, sweet taste receptors are expressed on pancreatic β-cells and take part in the regulation of insulin secretion [49,50]. Interestingly, in animal studies, Tas1R3−/− KO mice had reduced insulin sensitivity and glucose tolerance [51]. Moreover, reduced size of pancreatic islets and decreased density in the pancreatic tissue were observed [52]. Interestingly, these mice were characterized by increased cortical bone mass due to reduced osteoclast function [53].

### 3.2. Amino Acid-Sensing Receptors: Calcium-Sensing Receptor (CaSR), GPRC6A and GPR92

There are several other nutrient-sensing receptors outside the oral cavity, which take part in the detection of amino acids. Calcium-sensing receptor (CaSR) is a G protein-coupled receptor expressed on the surface of enteroendocrine cells. It can detect aromatic L-amino acids (phenylalanine, tryptophan, asparagine, and glutamine), and its activation leads to incretin secretion (GLP-1, GIP, and CCK). Divalent cations can activate this receptor. On the surface of absorptive enterocytes, CaSR can regulate intestinal calcium and other divalent absorption and anion secretion. It stimulates Cl^−^ and short-chain fatty acid-dependent HCO_3_^−^ secretion but inhibits cAMP-dependent HCO_3_^−^ secretion and modulates fibroblast growth factor-23 (FGF-23) production, which has a role in phosphate homeostasis and the gut–kidney–parathyroid gland axis. Moreover, L-amino acids could modulate calcium and other divalent cation homeostasis throughout the activation of CaSR. The calcium-sensing receptor is also expressed on myenteric plexi to modulate gut motility [54].

The calcium-sensing receptor plays a role in the regulation of inflammatory processes. In mice, CaSR regulates NLRP3 inflammasome via intracellular calcium release and cAMP [55]. In mice with dextran sulfate sodium (DSS)-induced colitis (an animal model of IBD), high protein intake caused an increased expression of inflammatory cytokines through the modulation of CaSR [56], while L-tryptophan, L-valine, and glutamyl dipeptides inhibited CaSR-dependent pro-inflammatory cytokine secretion in this colitis mouse model [57]. C57BL/6 intestinal Casr−/− KO mice had decreased expression of C-type lectin-encoding genes (Reg3b and Reg3g), which protects against Gram-negative and Gram-positive bacteria. Moreover, increased inflammatory protein secretion, increased expression of costimulatory molecules in colonic dendritic cells, increased Th1 and Th17 cell polarization, and increased FOXP3+ Treg cell number were observed in a compensatory way. These mice showed intestinal dysbiosis and enhanced susceptibility to DSS-induced colitis. However, the exact role of CaSR in the pathogenesis of human IBD is not known, so it needs further investigation [58]. Moreover, in colorectal cancer, CaSR expression is reduced and may have a preventative role in colorectal cancer development based on its role in the anti-inflammatory processes [59].

Interestingly, CaSR is expressed in human monocytes, and the expression level is positively correlated with severe coronary artery calcification in patients suffering from rheumatoid arthritis [60].

GPRC6A receptor detects L-arginine, L-lysine, and L-ornithine amino acids, but some divalent cations, testosterone, and osteocalcin can be activated in a tissue-specific manner. In an animal model study, Gprc6−/− KO mice had osteopenia, feminization, and metabolic syndrome. After being fed a high-fat diet for 25 weeks, these KO mice demonstrated significantly higher body weight, increased fat mass, and elevated plasma insulin and leptin levels than wild-type mice, but chow-fed KO mice did not show these abnormalities. GPRC6A may have a role in diet-induced obesity and the regulation of energy balance [56]. In a DSS-induced colitis mouse model, GPRC6A regulates colonic innate lymphoid cell 3 (ILC3) proliferation. ILC3 cells are localized in the lamina propria and play a role in immunomodulation, microbiota balance, and tissue repair to maintain gut homeostasis, as well as secrete IL-22. Stimulation with L-arginine leads to IL-22 production and ILC3 proliferation, and rapamycin inhibits this process [61].

The other receptor GPR92 is also expressed outside the oral cavity: on G-cells, it detects partially digested proteins and takes part in the secretion of gastrin [62].

### 3.3. Lipid-Sensing Receptors: Free Fatty Acid Receptors 1–4 (FFAR1–4)

Artificial sweeteners affect intestinal sweet taste receptors, intestinal peptide hormone, and insulin secretion, as well as changes in the microbiome. It could drive modified microbial metabolite production such as altered short-chain fatty acid (SCFA) composition. These fatty acids are ligands for other G protein-coupled receptors, including FFAR2 and FFAR3, which are expressed in the intestine, pancreatic β-cells, and innate immune cells (i.e., neutrophils, dendritic cells, macrophages, and mast cells), but not on lymphocytes. FFAR2 has a role in bacterial recognition and infection control, and the expression profile of neutrophils changes during sepsis.

Moreover, FFAR2 may have a pathogenic role in IBD development. Bacteria can produce SCFAs via different metabolic pathways. Acetate and butyrate are primarily produced via acetyl-Coenzyme-A, while propionate is produced via pyruvate or phosphoenolpyruvate through different pathways. Propionate and acetate are the main ligands of FFAR2, while butyrate primarily binds to FFAR3 [63]. Short-chain fatty acids could modify immune functions and drive GLP-1 and PYY release from enteroendocrine cells of the colon [64]. SCFAs maintain epithelial integrity and intestinal homeostasis and have antibacterial and anti-inflammatory functions, but they could also play a role in the activation of NLRP3 inflammasomes from colonic epithelial cells and IL-18 production. The role of FFAR3 in the immune response is not exactly known yet. Butyric acid and leucine can be induced via FFAR2/3 alpha-defensin secretion by Paneth cells, and in Crohn’s disease and obesity, alpha-defensin levels are decreased. Alpha-defensins can increase the polarization of FOXP3+ Tr cells [65,66].

Other G protein-coupled receptors, including FFAR1 and FFAR4 (GPR120) receptors, detect medium- and long-chain fatty acids, such as n-6 and n-3 PUFAs. The effects on GLP-1 secretion are controversial. FFAR-4 agonist ligands cause GLP-1 secretion in in vivo mouse models and in vitro human cells [67,68], but their major role in GLP-1 secretion cannot be confirmed in rats [69]. Agonisms of FFAR-4 improve insulin sensitivity and cause anti-inflammatory cytokine secretion in mice, and FFAR-4 interacts with some important signaling pathways. It acts directly on PPARγ to inhibit NF-κB, consequently leading to a decrease in the secretion of inflammatory cytokines such as TNF-α, IL-1, IFN-γ, IL-6, and IL-12 [70,71]. FFAR-4 takes part in the differentiation and activation of dendritic cells and influences the balance of Treg/Th17 and antiviral responses in mice [72].

Short-chain fatty acids have a receptor-independent effect in the inhibition of histone deacetylation [66]. FFAR-4 takes part in the pathogenesis of type 2 diabetes mellitus and colorectal cell cancer. It seems that a loss of expression of FFAR-4 is an early event in the progression of CRC [73]. 

In short, nutrient-sensing receptors take part in innate immunity, inflammation, regulation of the metabolic process, and feeding behavior. We summarized their various functions and health outcomes in a synoptic table (Table 2).

## 4. Effects of Artificial and Low-Calorie Sweeteners on Nutrient-Sensing Receptors, Gut Microbiota and Metabolism

### 4.1. Saccharin

The first artificial sweetener, saccharin (1,2-benzisothiazol-3-one-1,1-dioxide), was discovered in 1870. It was banned in 1970 by the FDA because saccharin mixed with cyclamate increased the incidence of bladder tumors in rats, but in 2001, saccharin was declared by the FDA to be non-carcinogenic with limited daily intake. Its sweetness strength is 200–300× that of sucrose. In terms of kinetics, approximately 85% to 95% of saccharin is absorbed by the small intestine, and its binding to plasma proteins is reversible. It is eliminated in urine, with its residual being excreted in feces. Saccharin can pass to the placenta, and its concentration in a fetus decreases more slowly than in maternal tissues [74,75].

Saccharin not only activates T1R2/T1R3 sweet taste receptors but also activates T2R43 and T2R44 bitter taste receptors at the same concentrations [76]. It is important that saccharin not only activates T1Rs in the oral cavity or gastrointestinal system but also in the whole body.

Findings about the effect of saccharin on insulin secretion and gut microbiota are controversial. In an in vitro study with pancreatic β cells from mice, saccharin-induced insulin secretion via T1R2/T1R3 stimulation directly in a TRPM5-dependent manner [77]. In mice with DSS-induced colitis, saccharin supplementation altered gut microbiota composition and reduced bacterial overload but did not affect intestinal permeability, and saccharin might have a protective effect on intestinal inflammation [78].

In a mouse and human nutritional study, saccharin consumption led to dysbiosis and increased glucose intolerance. In mice, 11-week oral consumption of saccharin, sucralose, or aspartame enhanced glucose intolerance, which was mediated by an altered microbiota composition [79]. However, in another mouse and human study, saccharin did not affect gut microbiota composition and insulin sensitivity [80].

### 4.2. Sucralose

Sucralose (1,6-dichloro-1,6-dideoxy-β-D-fructofuranosyl-4-chloro-4-deoxy-α-D-galactopyranoside) is a chemically modified sucrose, and its sweetness potency is 385- to 650-fold greater than that of sucrose. Sucralose is not absorbed into the body or metabolized for energy and does not affect blood glucose levels. It is excreted in feces (99%) and in urine (1–2%). It is unknown if sucralose crosses the placental transfer or passes through the blood-brain barrier [74].

During the manufacturing process, sucralose-D-acetate is generated as an intermediate product, but this molecule could be formed in the intestine via acetylation. Sucralose-D-acetate is genotoxic, damages the intestinal tight junctions, and significantly increases the expression of inflammatory genes. Further safety investigations are needed based on these findings from a previous study [81]. In an animal model, SAMP mice (which naturally develop segmental enteritis with cobblestone lesion formation in a fashion resembling Crohn’s disease) fed with Splenda (sucralose with maltodextrin) had increased myeloperoxidase (MPO) activity, but not in IBD-free mice, while dysbiosis (Proteobacteria enrichment) was observed in the control group [82]. In an animal model of colorectal cancer involving dextran sulfate sodium (DSS)-induced colitis mice treated with azoxymethane (AOM), sucralose caused significantly increased levels of inflammatory cytokines (TNF-alpha and IL-1ß), decreased IL-10, increased size of the spleen, altered the gut microbiota composition and gut barrier function, and increased the number and size of colorectal tumors [83]. In contrast, in another animal model study, daily sucralose consumption for 8 weeks not only positively affected the body weight but also the glucose metabolism of mice fed a high-fat diet (HFD). However, in the control group, other metabolic parameters were unchanged [84]. In C57BL/6 mice fed a HFD, sucralose consumption accelerated hepatic steatosis and lipid accumulation, which induced reactive oxygen species in a T1R3-dependent manner. The expression level of T1R3 was unchanged after sucralose consumption, but this effect was inhibited with the use of silencing RNA and lactisole based on in vitro and in vivo investigations [85].

Interestingly, high-dose sucralose consumption in mice modulates T-cell proliferation and their effector functions. Sucralose treatment leads to reduced intracellular calcium release of T cells and decreased PLC-gamma activation, but there are no changes in other types of lymphocytes [86].

In an in vitro model of the retinal epithelium, sucralose treatment reduced vascular endothelial growth factor (VEGF), the principal mediator of diabetic and proliferative retinopathy-induced vasculogenic processes, in a T1R3-dependent manner [87].

In a human study, daily oral sucralose consumption for 4 weeks decreased insulin sensitivity and GLP-1 secretion in healthy volunteers [88]. In another study, daily consumption of 48 mg of sucralose for ten weeks altered the gut microbiota by increasing the number of Blautia coccoides and decreasing the number of Lactobacillus acidophilus in healthy, non-insulin-resistant young adults [89].

### 4.3. Acesulfame Potassium

Acesulfame potassium (ACK) contains sulfonamide, which exerts an antimicrobial activity to decrease glucose fermentation by intestinal bacteria. In an animal study, C57BL/J6 mice that were administered ACK showed increased proinflammatory cytokine secretion (TNF, IL-1ß, and INF-gamma), but decreased GLP-1R and GLP-2R expression, and ACK-induced small intestine injury with increased intestinal permeability, as well as increased migration of lymphocytes to the intestinal microvessels. Dysbiosis was observed in the ACK-treated mice, and this injury was not transferred to recipient mice with fecal transplantation. Previously, ACK treatment enhanced the secretion of both GLP-1 and GLP-2, and the downregulation of their receptors could provide negative feedback, but the exact mechanism is not yet known. Notably, in this study, the dosage of ACK was higher than the dosage used in humans [90]. In CD-1 mice, consumption of ACK for 4 weeks caused weight gain in male mice, but not in female mice. Moreover, ACK changed the gut microbiota composition in a gender-specific manner [91].

### 4.4. Aspartame

Aspartame, another artificial sweetener, has the same caloric content per gram as sucrose but is more than 200 times sweeter. Aspartame is a combination of the amino acids l-phenylalanine and l-aspartic acid, which are connected through methyl ester bonds. Aspartame is disintegrated into methanol, aspartic acid, and phenylalanine, and it does not reach the general circulation as an intact molecule. Its amino acid components are converted in enterocytes to oxaloacetate via transamination before reaching the portal circulation and entering the free amino acid pool (as methionine, threonine, isoleucine, and lysine) to participate in the urea cycle and gluconeogenesis. The other amino acid component, phenylalanine, is converted to tyrosine, which could be converted into neurotransmitters, including dopamine, norepinephrine, and epinephrine [74].

The findings of existing in vitro, in vivo, and RCT studies are conflicting about the effect of aspartame on insulin sensitivity and microbiome alteration. In animal studies, chronic aspartame consumption increased the levels of fasting glucose in both the control and high-fat groups independently of body composition. Moreover, aspartame was rapidly metabolized in relation to propionate production and affected the gut microbiota composition; Enterobacteriaceae and Clostridium leptum abundance was increased [5,92]. Consumption of aspartame for 12 weeks did not affect glucose metabolic parameters, appetite, and body weight in a randomized nutritional study with 100 healthy participants [93]. The effect on CaSR receptors is not known yet.

A number of studies have investigated the effects of aspartame on several diseases. Aspartame may have a genotoxic effect, amyloidogenic properties, and influences on behavior and mental stress [94], but its consumption may not trigger autism [95]. Aspartame could elevate the serum corticosterone and adrenocorticotropic hormone levels [94].

There are studies on the connection of aspartame to carcinogenesis. In one study, increased prenatal exposure to aspartame was associated with an increased prevalence of malignancies in children and an increased prevalence of non-Hodgkin lymphoma in adults [96]. In contrast, in another cohort study, the prevalence of multiple myeloma was not associated with aspartame and sugar-sweetened beverage consumption [97].

### 4.5. Other Studies with Artificial Sweeteners (Cohort, Clinical and Animal)

In an observational study with 232 participants, regular AS (saccharin, ACK, sucralose, and aspartame) intake for 7 days led to significantly increased GIP secretion over 2 h via T1R3 sweet taste receptors. There were no significant differences in fasting glucose, insulin, and GIP levels among LCS and non-LCS users. In this study, food diaries and frozen blood samples were collected from the Baltimore Longitudinal Study of Aging (BLSA) participants, who underwent an oral glucose tolerance test (OGTT) and had no diabetes [98], and analyzed for glucose, insulin, and GIP levels.

In an in vitro study with the intestinal epithelial Caco-2 cell line, treatment with aspartame, sucralose, and saccharin had an effect on permeability. At high doses, saccharin and aspartame induced cell death and apoptosis, and aspartame also caused elevated ROS production via T1R3 activation. Moreover, sucralose and aspartame at lower concentrations caused disruption of the intestinal barrier function [99].

Three artificial sweeteners (aspartame, saccharin, and sucralose) were investigated and had differential effects on the pathogenicity of *E. coli* and *E. faecalis* [100]. Moreover, four commonly used non-nutritive sweeteners (saccharin, aspartame, acesulfam K, and sucralose) promote the horizontal transfer of antibiotic-resistance genes via conjugation in both environmental and clinical settings. Acesulfam K (ACK) was found to trigger reactive oxygen production in E.coli, but not in Acinetobacter bayli [101]; it is possible that different bacterial species could react in a different manner to this AS. In another investigation, ACK had potent anti-biofilm activity at sub-inhibitory concentrations and disrupted biofilms. Sub-lethal concentrations of ACK increased the sensitivity of *A. baumannii* to a number of antibiotics, particularly to carbapenems [102].

Interestingly, in an animal (rat) study, regular consumption of ACK in early life led to a change in sugar-motivated behavior, glucoregulation in adulthood, sweet taste receptor expression, and genetic alterations associated with collagen synthesis in the hippocampus, which produced hippocampal-dependent memory dysfunction in later life [103].

In the French population-based prospective cohort NutriNet-Santé study (*n* = 102,865 adults with a median follow-up of 7.8 years), the effects of consumption of artificial sweeteners were suggested to increase overall cancer risk (especially aspartame and acesulfame K). Aspartame intake had a positive correlation with breast and obesity-related cancer development [104].

Another investigation based on the French population-based NutriNet-Santé study (*n* = 155,588 adults with a median follow-up of 9.13 years) reported a significant correlation between artificial sweetener consumption (aspartame and ACK) and a higher risk for type 2 diabetes mellitus, but the association with sucralose intake was not significant in the sensitivity analyses [105]. Moreover, the risk of cardiovascular diseases in the NutriNet-Santé cohort (*n* = 103,388 adults with a median follow-up of 9.0 years) was significantly higher with the consumption of artificial sweeteners. There was also a direct association between sucralose consumption and the risk of angioplasties. There was a significant positive correlation between aspartame-sweetened beverages and coronary heart disease risk [106]. In another analytical cohort study based on the Women’s Health Initiative Observational Study (*n* = 81,714 women with a mean follow-up of 11.9 years), regular consumption of artificial sweeteners was associated with a significantly higher risk of all-cause mortality, especially stroke, small artery occlusion, and coronary heart disease [107].

Artificial sweeteners interact with sweet and bitter taste receptors, and based on the findings of several studies, they have an effect on intestinal permeability, oxidative stress, and cell proliferation through these receptors. They may have effects on the pathogenesis of some diseases, including increasing the risk of development of various malignancies, type 2 diabetes mellitus, and cardio- and cerebrovascular diseases, but the findings of existing studies are controversial [92,98,99,104].

There are studies with conflicting results regarding the influences on the gut microbiota composition [108] and the abundance of *Bacteroides* species has been reported to increase after saccharin treatment [79]. The bacteriostatic effect of saccharin could be beneficial to intestinal inflammation by decreasing the bacterial load based on animal models, but further long-term human studies are needed to understand the effects related to changes in the microbiome composition [108]. The findings of these studies could be important because IBD patients prefer artificial sweeteners, juice, and soda, believe that artificial sweeteners have no effect on health, and eat fewer vegetables and fruits [109]. However, based on a small, one-sample study, the consumption of artificial sweeteners could be harmful, especially to patients suffering from IBD and hepatic steatosis, as it may promote disease progression. Aside from this, dysbiosis could influence glucose intolerance and incretin secretion and may have innate immunological effects.

### 4.6. Steviol Glycosides and Sugar Alcohols

There are other sweeteners that are technically not artificial sweeteners, such as steviol glycosides and sugar alcohols, but have an effect on sweet taste receptors.

Steviol glycosides are derived from the plant Stevia rebaudiana Bertoni. Four major and at least six less prevalent steviol glycosides have been isolated. The most important glycosides are stevioside and rebaudioside A, which are hydrolyzed to steviol in the colon and can be absorbed [74].

In one study, stevia did not significantly change the blood glucose, insulin, and HbA1c levels or body weight after 8 weeks of daily consumption among type 2 diabetes mellitus patients [110]. However, in another study, Stevia consumption significantly decreased postprandial glucose levels compared to sucrose [111].

In an animal model study, daily intake of stevioside prevented the development of high-fat-diet-induced diabetic hyperglycemia in wild-type mice, but not in Trpm5−/− mice. The TRPM5 cation channel is essential for the biological effects of steviol glycosides and functions independently of T1R2/T1R3 receptors [112]. Steviol glycosides may have beneficial effects on blood glucose metabolism, but further studies are needed [113]. In an in vitro study, rebaudioside A stimulated GLP-1 and PYY secretion in enteroendocrine cells from the small intestine of mice and increased enteroendocrine cell number in two-dimensional cell culture [114]. Stevia extracts had an effect on cytokine secretion in vitro, and it could decrease the secretion of TNF-alpha, IL-6, and IL-1ß. In an animal model involving dextran sulfate sodium-induced colitis mice, Stevia inhibited the activation of NFKB and MAPK signaling and might mimic probiotic action. It could modify the microbiome of the intestine, but the results are controversial [115] and further studies are needed.

Sugar alcohols are used as low-calorie or non-nutritive sweeteners. Erythritol is present naturally in some fruits such as grapes and watermelon. The sweetness of erythritol is only 70% of that of sucrose. Erythritol is absorbed in the small intestine, but a small amount of it is fermented by bacteria in the colon, whereas the rest is excreted in urine. Erythritol reduces postprandial glucose by inhibiting alpha-glucosidases, stimulating GLP-1, PYY, and CCK secretion, and delaying gastric emptying [116]. In another study, lactisole could not inhibit incretin secretion, which means that its action could not be mediated via the T1R2/T1R3 receptors of the intestine [117]. Erythritol participates in metabolism, and it can affect SCFA production to increase butyrate and propionate concentration. There is endogenous erythritol synthesis from glucose or fructose via the pentose phosphate pathway, which is catalyzed by alcohol dehydrogenase 1 and sorbitol dehydrogenase, and NADPH is a required cofactor. Elevated serum levels of erythritol are associated with type 2 diabetes mellitus, central adiposity gain, metabolic syndrome, and elevated risk of cardiovascular diseases [118,119]. In an animal study, the consumption of non-nutritive sweeteners (99% erythritol + 1% aspartame) for 4 weeks resulted in significantly greater visceral adiposity, increased adipocyte cell size, and increased leptin expression compared to the control mice. On the other hand, in C57BL/6J mice fed a high-fat diet, the consumption of water containing 5% erythritol resulted in significantly reduced body weight, decreased glucose intolerance, and higher metabolic rate than the control mice [120]. In vitro, erythritol modulates the polarization of macrophages to the M1 phenotype and increases the production of proinflammatory cytokines [121]. Further studies are needed on the effects of chronic erythritol consumption on metabolism and inflammation and the relation to endogenous erythritol synthesis.

Xylitol is a five-carbon sugar alcohol, which occurs in fruits, vegetables, mushrooms, seaweed, and yeast. Xylitol inhibits the growth of Escherichia ssp. but enhances Bifidobacteria and *Bacteroides* ssp. growth. However, the findings of existing investigations are conflicting; *Bacteroides* ssp. growth was inhibited by xylitol in some studies. Propionate production is increased via the pentose phosphate pathway, which means xylitol could modulate SCFAs and the microbiome in the gut, but further investigations are needed [122]. Xylitol is incompletely absorbed, and the majority is fermented by bacteria in the colon. Potential adverse events are abdominal discomfort, bloating, and diarrhea due to the osmotic effect. Absorbed xylitol is converted in the liver to xylose, which is phosphorylated to xylulose-5-phosphate, an intermediary product of the pentose phosphate pathway, and, finally, converted to glucose. Xylitol has a dose-dependent effect on the release of CCK, GLP-1, and PYY incretin hormones. It also causes decreased gastric emptying and increases the uric acid level acutely, but it has no effect on the GIP, insulin secretion, motilin, and blood lipid profile [123]. After treatment with intragastric loads of 50 g of xylitol, 75 g of erythritol, or 75 g of glucose dissolved in 300 mL of tap water, xylitol was observed to increase cerebral blood flow in the hypothalamus, glucose had the opposite effect, whereas erythritol had no effect [124]. However, it is not known how sweet and bitter taste receptors are involved in the metabolic and neurological regulation processes.

Maltitol, another low-calorie sugar alcohol that is derived from maltose via hydrogenation, could bind to the T1R2/T1R3 receptor complex and the T1R2 binding site [125]. Its sweetness and taste are comparable to sucrose. Maltitol has a slow absorption rate and is metabolized by colonic bacteria. It increases SCFAs and acts as a prebiotic that increases Bifidobacteria, but further research is needed to determine its exact role in the modulation of the microbiome [5]. Maltitol infusion to the terminal ileum of dogs increases GLP-1 secretion [126], but the effect is absent in humans. Maltitol inhibits glucose absorption from the isolated jejunum of rats ex vivo, but not in vivo [127].

Irritable bowel syndrome (IBS), which is a functional gut disorder with slight inflammatory processes, disrupts the brain–gut axis and intestinal permeability. The main symptoms are bloating, recurrent abdominal pain, and changes in stool frequency and form. This gastrointestinal disease is one of the most common among patients across the world. A diet with low fermentable oligosaccharides, disaccharides, monosaccharides, and polyols (FODMAPs) could help decrease the symptoms. As mentioned above, polyols may have a probiotic effect by increasing SCFA contents and modulating the microbial composition when consumed in small doses. Polyols could trigger gastrointestinal syndromes in IBS: after polyol ingestion malabsorption, there was evidence of greater bowel dysmotility and intestinal hypersensitivity in patients with IBS than in healthy controls [128]. Moreover, the usage of a low-FODMAP diet decreased IBS-like symptoms and inflammatory markers (CRP, stool calprotectin) and enhanced the quality of life of patients with IBD [129]. Further studies are needed on the exact effect on the gut microbiota and metabolism not only in healthy individuals but also in patients with gastrointestinal disorders.

## 5. Discussion

Nutrient-sensing receptors have a complex role in the gastrointestinal system, including the modulation of gastric emptying, nutrient and ion absorption, and secretion, glucose metabolism via the regulation of incretin hormone and insulin secretion, and several inflammatory processes [31,49,54]. Naturally, nutrient-sensing receptors are expressed outside of the gastrointestinal system. They are also expressed in the airway system and on immune cells throughout the body to take part in the regulation of innate and adaptive immune processes, as well as in the peripheral and central nervous system and on the cells of the placenta [15,18,21,22,25].

These intestinal G protein-coupled taste receptors provide crosstalk between dietary components, toxic substances, drugs, ion intake, and the regulation of the immune, endocrine, and neurological processes [10,16,21,46]. Altered intestinal microbiota composition could modulate these receptors. Thus, they play a role in the modulation of nutritional behavior. Changes in microbial components by means of dietary constituents could determine not only the direction of immune responses via the secretion of several cytokines but also the modulation of metabolism via incretin hormone secretion [63,64].

High-fat and high-sugar consumption is part of the Western diet and leads to inflammatory processes. On the other hand, the usage of artificial and low-calorie sweeteners, instead of sugar intake, can modulate intestinal microbiota, nutrient-sensing receptors, incretin secretion, and inflammation [1,2,130]. Artificial sweeteners may affect the antibiotic resistance mechanism of pathogens and the gut microbiota [101,102].

Based on the findings of previous studies, sweeteners may have a role in the pathogenesis of metabolic syndrome, inflammatory bowel diseases, and irritable bowel syndrome, as well as the evolution of gastrointestinal and other malignancies, via the modulation of nutrient-sensing receptors [83,95,96,104,105,106,107]. Moreover, because of the expression pattern of nutrient-sensing receptors and the complexity of the gastrointestinal system, their effects on nutrient-sensing receptors will be systemic.

## 6. Future Directions

Further studies are needed on the exact role of nutrient-sensing receptors in health and diseases. Nutrient-sensing receptors have a central role in the regulation of the gastrointestinal tract (hormonal, metabolic, and neurological). These pathways of intestinal nutrient-sensing receptors could be a potential therapeutic target in some metabolic and inflammatory diseases. Further investigations are needed on the effects of artificial sweeteners on the gut microbiota, antibiotic resistance, insulin and glucose metabolism, innate immunity, and the development of some diseases, e.g., cancer, metabolic syndrome, irritable bowel syndrome, and inflammatory bowel diseases.

## 7. Conclusions

The findings of previous studies are controversial, and for all sweeteners, further studies are needed to explore the exact mechanisms underlying their effects on pathogenesis and their potential clinical role in many diseases. Moreover, to complicate the situation, there are only animal or in vitro studies available regarding some effects and mechanisms, and, for some human clinical studies, their small sample size is a limiting factor. It is difficult to know what the risk/benefit ratio actually is, and new knowledge can help answer the question. The place of artificial sweeteners will be established sooner or later, as the interests and the health of consumers are always paramount. The more knowledge we gather about their mechanisms and effects, the more certain we are that there are other consequences in exchange for the sweet taste.

## Figures and Tables

**Table 1:** Taste receptors and their ligands of enteroendocrine cells and changes incretin hormone secretion in IBD patients [9,10,11,12,13,14].

Enteroendocrine Cell Type	Localization	Secreted Hormones	Taste Receptors	Serum Hormone Level Changes during IBD
X/A-cells	stomach	ghrelin, nesfatin1	sugars, amino acids bitter LCFAs T1R3 T2R FFAR4 CaSR	elevated
G-cells	stomach	gastrin	amino acids, sugars peptides T1R3 bitter T2R CaSR GPRC6A GPR92 (LPAR5)	elevated
P-cells	stomach	leptin		N/A
Enterochromaffin-like cells	stomach	histamine		N/A
D-cells	stomach, small intestine	somatostatin		N/A
I-cells	small intestine	CCK	Amino acids sugars, bitter, LCFA T1R1/T1R3 T2R CaSR GPR6C GPR120	
K-cells	small intestine	GIP		
L-cells	small intestine, colon	GLP-1, GLP-2	Sugars, bitter, SCFAs, LCFAs amino acids T1R2/T1R3, T2R FFAR 1/2/3 GPR120 GPR6C	elevated
Enterochromaffin cells	colon	5-HT		decreased

LCFA: long-chain fatty acid, SCFA: short-chain fatty acid, GIP: glucose-dependent insulinotropic polypeptide; GLP-1: glucagon-like peptide-1; GLP-2: glucagon-like peptide-2, 5-HT: 5-hydroxytryptamine, CaSR: Ca^2+^-sensing receptor, FFAR 1–4: Free fatty acid receptor 1–4, GPRC6A: G protein-coupled receptor class C group 6 member A, GPR92 (LPAR5): lysophosphatidic acid receptor 5, IBD: inflammatory bowel diseases, N/A: not applicable

**Table 2:** Summary on the role of nutrient sensing receptors.

Receptor	Ligand	Expression	Function	Health Outcome
Sweet taste receptors i.e., T1R3	sugars saccharin sucralose aspartame acesulfame K amino acids Na-glutamate	HGE neurons (brain) solitary chemosensory cells (upper airway system) chemosensory brush cell (urinary system) neutrophil granulocytes T and B lymphocytes Enteroendocrine cells Tuft cells Paneth cells Pancreas ß-cells	glucose metabolism, blood-brain axis regulation host-pathogen interaction cell migration cell activation incretin secretion glucose absorption Th2 immunity regulation antimicrobial peptides secretion insulin secretion	feeding behavior circadian rhythm regulation allergic, infectious diseases, chronic rhinosinusitis innate immunity glucose metabolism, metabolic syndrome IBD, helminth and viral infections, inflammation IBD infections, inflammation diabetes mellitus
Bitter taste receptors i.e., T2R38	drugs i.e., chloroquine saccharin acesulfame K sucralose bacterial peptides: i.e., acyl-homoserine lactones	placenta myeloid cells macrophages chemosensory cells (upper airway system) chemosensory brush cell (urinary system) Enteroendocrine cells Goblet cells Paneth cells	unknown migration phagocytosis production of antimicrobial peptides glucose metabolism regulation mucin secretion antimicrobial peptide secretion	unknown innate immunity infection, inflammation chronic rhinosinusitis diabetes mellitus, metabolic syndrome, inflammation inflammation, infections inflammation, infections
CaSR	aromatic L-amino acids	enteroendocrine cells	calcium homeostasis cytokine secretion	calcium homeostasis gut–kidney axis inflammation, IBD? cancer development
GPRC6A	amino acids L-arginine, L-lysine and L-ornithine osteocalcin testosterone	enteroendocrine cells ILC-3 cells	bone metabolism IL-22 secretion tissue repair microbiota balance	bone resorption inflammation, IBD
GPR92 (LPAR5)	partially digested proteins	G cells	gastrin secretion	digestion regulation
FFAR 1, 4	n-6 and n-3 PUFAs, DHA	enteroendocrine cells lymphocytes dendritic cells macrophage	antiinflammatory cytokine secretion Treg/Th17 axis regulation insulin sensitivity	inflammation innate immunity antiviral response diabetes mellitus
FFAR 2,3	short-chain fatty acid	enteroendocrine cells innate immune cells: neutrophil granulocytes pancreatic ß cells	epithelial integrity antiinflammatory cytokine secretion NLRP3 inflammasome modulation alpha defensin secretion glucose metabolism	gut permeability microbiome regulation inflammation IBD diabetes mellitus infection diabetes mellitus

HGE neurons: high glucose-excited neurons, IBD: inflammatory bowel diseases, IL-22: interleukin-22, ILC-3: innate lymphoid cells-3, CaSR: Ca^2+^-sensing receptor, FFAR 1-4: Free fatty acid receptor 1–4, GPRC6A: G protein-coupled receptor class C group 6 member A, GPR92 (LPAR5): lysophosphatidic acid receptor 5, PUFA: polyunsaturated fatty acid, DHA: Docosahexaenoic acid, NLRP3: NLR family pyrin domain containing 3, Th2: T helper 2.
